# Enzymatic debranching is a key determinant of the xylan-degrading activity of family AA9 lytic polysaccharide monooxygenases

**DOI:** 10.1186/s13068-022-02255-2

**Published:** 2023-01-05

**Authors:** Monika Tõlgo, Olav A. Hegnar, Johan Larsbrink, Francisco Vilaplana, Vincent G. H. Eijsink, Lisbeth Olsson

**Affiliations:** 1 Division of Industrial Biotechnology, Department of Biology and Biological Engineering, Chalmers University of Technology, 412 96 Gothenburg, Sweden; 2 Wallenberg Wood Science Centre, Chalmers University of Technology, 412 96 Gothenburg, Sweden; 3 Faculty of Chemistry, Biotechnology and Food Science, NMBU-Norwegian University of Life Sciences, 1433 Ås, Norway; 4 Division of Glycoscience, Department of Chemistry, KTH Royal Institute of Technology, 106 91 Stockholm, Sweden; 5 Wallenberg Wood Science Centre, KTH Royal Institute of Technology, 100 44 Stockholm, Sweden

**Keywords:** Lytic polysaccharide monooxygenase, LPMO, Arabinofuranosidase, Glucuronidase, Lignocellulolytic cocktail, Plant cell wall, Lignocellulose, Cellulose, Xylan

## Abstract

**Background:**

Previous studies have revealed that some Auxiliary Activity family 9 (AA9) lytic polysaccharide monooxygenases (LPMOs) oxidize and degrade certain types of xylans when incubated with mixtures of xylan and cellulose. Here, we demonstrate that the xylanolytic activities of two xylan-active LPMOs, *Tt*LPMO9E and *Tt*LPMO9G from *Thermothielavioides terrestris*, strongly depend on the presence of xylan substitutions.

**Results:**

Using mixtures of phosphoric acid-swollen cellulose (PASC) and wheat arabinoxylan (WAX), we show that removal of arabinosyl substitutions with a GH62 arabinofuranosidase resulted in better adsorption of xylan to cellulose, and enabled LPMO-catalyzed cleavage of this xylan. Furthermore, experiments with mixtures of PASC and arabinoglucuronoxylan from spruce showed that debranching of xylan with the GH62 arabinofuranosidase and a GH115 glucuronidase promoted LPMO activity. Analyses of mixtures with PASC and (non-arabinosylated) beechwood glucuronoxylan showed that GH115 action promoted LPMO activity also on this xylan. Remarkably, when WAX was incubated with Avicel instead of PASC in the presence of the GH62, both xylan and cellulose degradation by the LPMO9 were impaired, showing that the formation of cellulose–xylan complexes and their susceptibility to LPMO action also depend on the properties of the cellulose. These debranching effects not only relate to modulation of the cellulose–xylan interaction, which influences the conformation and rigidity of the xylan, but likely also affect the LPMO–xylan interaction, because debranching changes the architecture of the xylan surface.

**Conclusions:**

Our results shed new light on xylanolytic LPMO9 activity and on the functional interplay and possible synergies between the members of complex lignocellulolytic enzyme cocktails. These findings will be relevant for the development of future lignocellulolytic cocktails and biomaterials.

**Supplementary Information:**

The online version contains supplementary material available at 10.1186/s13068-022-02255-2.

## Background

Polysaccharide-degrading lytic polysaccharide monooxygenases (LPMOs) are metalloenzymes whose activity depends on reduction of a single Cu atom in the catalytic center and an oxygen co-substrate (H_2_O_2_, O_2_) [1–4]. LPMOs belonging to Auxiliary Activity family 9 (AA9, [5]), LPMO9s, oxidize their cellulosic or hemicellulosic substrates either at C1, resulting in the formation of lactones that are in equilibrium with aldonic acids, or at C4, resulting in the formation of 4-ketoaldoses that are in equilibrium with gemdiols. Some LPMO9s produce mixtures of C1- and C4-oxidized products and may thus generate double oxidized products, whereas in some cases, oxidation at C6 has also been observed [6–10]. LPMOs were discovered in 2010 [1] and have since gathered wide interest in the industrial biotechnology field, due to their ability to significantly boost the activity of cellulases in the cellulolytic enzyme cocktails that are used to convert carbohydrates in renewable biomass to fermentable sugars for production of fuels and chemicals [11, 12]. The role of LPMO9s in saccharification of cellulose has been studied in much detail, as reviewed in e.g. [4] and [12]. It is becoming increasingly clear that certain LPMO9s, next to acting on linear and crystalline cellulose, can also act on one or more hemicelluloses, such as xyloglucan, glucomannan and arabinoxylan, either alone or when adsorbed to cellulose [8, 13–17]. Notably, these hemicelluloses are heteropolymers, often with substitution groups on the main chain, which raises interesting questions regarding the substrate specificity of LPMO9s and their interplay with other enzymes involved in hemicellulose debranching and depolymerization.

Secondary plant cell walls, the major constituents of plant biomass, are primarily comprised of cellulose, various hemicelluloses, and lignin. Their chemical nature and supramolecular structure make plant cell walls strong biocomposites that are resistant to chemical, biotic, and physical stresses. The recalcitrance of cellulose comes from its (largely) crystalline structure that is stabilized by numerous hydrogen bonds, hydrophobic interactions, and Van der Waals forces. Hemicelluloses share with cellulose a backbone of β-(1,4)-linked monosaccharides but lack a crystalline lattice and are heteropolymeric with numerous different monosaccharide building blocks. In addition, hemicelluloses show large variation in molecular composition and structure between plants and even tissues [18, 19]. Hemicelluloses referred to as xylans are comprised of a backbone of β-1,4-linked xylopyranosyl monomers that are often substituted by decorating sugars such as arabinose and/or (methyl)glucuronic acid ((Me)GlcA) and may carry acetyl groups [18–20]. In accordance with their predominating substitutions, xylans are often referred to as glucuronoxylan (GX), arabinoxylan (AX) or arabinoglucuronoxylan (AGX).

Although further corroboration is needed, it is known that xylans have a tethering function in plant cell walls by adsorbing to cellulose, and that adsorption strength depends on both the degree and pattern of substitution. The occurrence of regular motifs of glycosyl substitutions and acetylations has been reported for xylans from angiosperms [21–24] and gymnosperms [25, 26], and these motifs are thought to play an important role in modulating the interaction of hemicelluloses with cellulose and lignin [24, 27–29]. Current data indicate that adsorption is strongest for xylans that have a low degree of substitution [30–32] or a regular substitution pattern [23, 26, 27]. Xylans with no or very few substitutions tend to aggregate to each other and/or bind strongly to cellulose [31–33]. It has been shown that xylan adsorption to cellulose also depends on key properties of the latter, such as cellulose crystal geometry, surface area, porosity, and crystallinity [26, 30, 34].

Studies of LPMO9s from *Neurospora crassa* [13, 16], *Myceliophthora thermophila* [14, 35], *Podospora anserina* [15], *Malbranchea cinnamomea* [8] and *Thermothielavioides terrestris* [36] have revealed activity of these enzymes on hemicelluloses, such as xyloglucan from tamarind seeds, glucomannan from konjac, glucuronoxylan from beech and birchwood, and oat spelt arabinoxylan. In the case of xylans, LPMO9 activity, i.e., reductant-dependent generation of oxidized products, has only been convincingly demonstrated when using reaction mixtures that also contain cellulose. This dependency on the presence of cellulose has been explained [8, 14, 16, 36] by an expected conformational change of the xylan, which in solution adopts a threefold helical conformation but in combination with cellulose adopts a more stretched twofold helical structure that allows strong adsorption to cellulose [25–28, 37]. The resulting flatter surface of the xylan–cellulose co-polymeric structure seems well-adapted to the flat substrate-binding surfaces of xylan-active LPMO9s and likely mimics the ultrastructure of these polysaccharides in native plant cell walls.

*T. terrestris* is an extremophilic ascomycetous filamentous fungus that encodes and utilizes a plethora of lignocellulose active enzymes, including numerous AA9 LPMOs [38, 39]. In a previous study, we have characterized six LPMO9s from *T. terrestris* LPH172, three of which showed activity toward beechwood, birchwood and/or spruce xylans, only when these xylans were combined with phosphoric acid-swollen cellulose (PASC) [36]. Interestingly, our study revealed major substitution-dependent differences between the xylan-active LPMOs. For example, *Tt*LPMO9E showed activity on both acetylated and non-acetylated glucuronoxylan, but not on arabinoglucuronoxylan, whereas *Tt*LPMO9G showed (weaker) activity on non-acetylated glucuronoxylan and on arabinoglucuronoxylan [36]. None of these xylan-active LPMOs showed activity on wheat arabinoxylan (WAX), in line with early observations by Frommhagen et al. [14] who concluded that xylan-active *Mt*LPMO9A did not act on this highly substituted xylan type (WAX that had a 62:38 xylose:arabinose ratio).

Taken together, the considerations and observations described above lead us to the hypothesis that the activity of LPMO9s on xylan may be modulated by debranching enzymes, such as (methyl)-glucuronidases and arabinofuranosidases, because debranching will both modulate the crucial formation of LPMO-susceptible xylan–cellulose complexes and affect the ability of the LPMO to interact with the xylan. Therefore, we set out to investigate whether LPMO9 activity on a variety of xylans (WAX, spruce arabinoglucuronoxylan (SpAGX), beechwood glucuronoxylan (BeGX)) is affected by the action of a GH62 α-arabinofuranosidase and/or a GH115 (methyl)-glucuronidase. The results show that, generally, debranching leads to increased xylanolytic activity of LPMO9s and in some cases enables LPMO9 activity on substrates for which previously no activity had been detected. We also show that the xylanolytic activity of LPMO9s depends on the type of cellulose present in the reaction mixture. Next to underpinning the (known) impact of debranching enzymes on cellulose–xylan interactions, our results support the notion that AA9 LPMOs may play a significant role in xylan degradation that may depend on the interplay with debranching enzymes. Such interplay, as well as the newly discovered activities of LPMO9s on xylans as such, will likely be relevant in the development of future lignocellulolytic enzyme cocktails for biomass processing.

## Results

### The activity of *Tt*LPMO9E on WAX is inhibited by arabinosyl substitutions

**Fig. 1:**
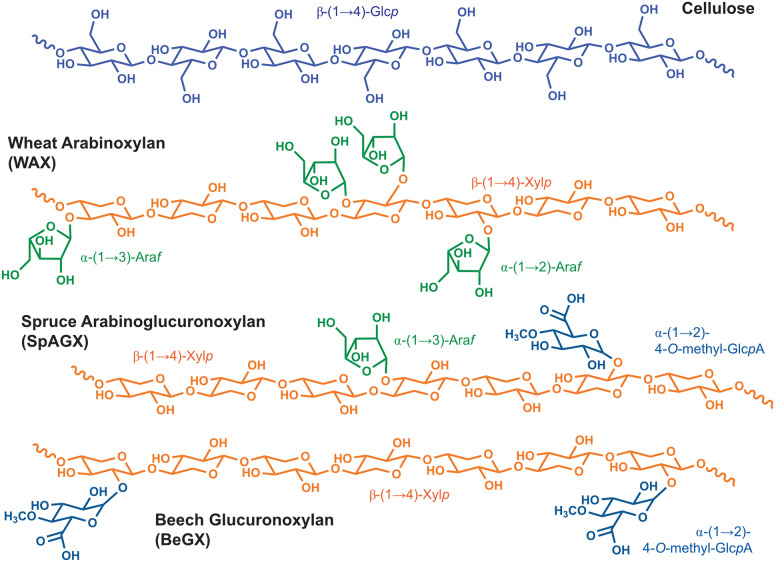
Chemical structures of the substrates used in this study. Note that in BeGX the glucuronic acid residues may or may not be methylated and the figure only shows the methylated form. The SpAGX used in this study contained mainly methylated glucuronic acid [40]. The GH115 used can remove both MeGlcA and GlcA [41]

We have previously shown that *Tt*LPMO9E is active on various xylan substrates but not on standard WAX, which is a highly substituted arabinoxylan with 38% of its monosaccharides being arabinose [36]. Next to this substrate, here referred to as WAX38 (38:62 Ara:Xyl; A/X ratio 0.61), we also tested WAX30 (30:70 Ara:Xyl; A/X ratio 0.43) and WAX22 (22:78 Ara:Xyl; A/X ratio 0.28), to assess whether reduced substitution could increase LPMO activity. Figure 1 shows the chemical structure of WAX and of the other xylan substrates (see below) used in this study.

**Fig. 2:**
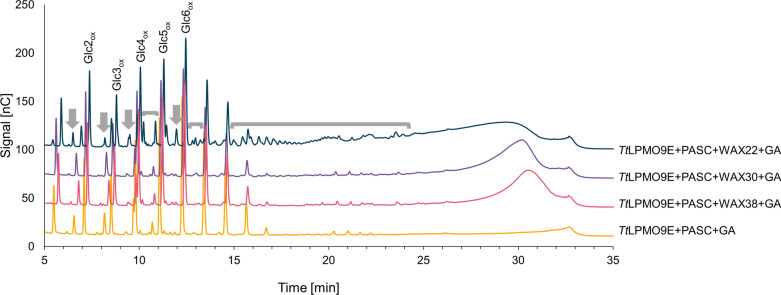
HPAEC–PAD chromatograms showing soluble products generated upon incubation of *Tt*LPMO9E with PASC, or a combination of PASC and differently substituted WAX at pH 6.5 and 40 °C, for 16 h. All reactions contained gallic acid (GA) as reductant. Oxidized cello-oligosaccharides resulting from PASC degradation are labelled as “GlcX_ox_”, where X indicates the degree of polymerization. Regions that show products resulting from LPMO-catalyzed degradation of WAX22 are indicated by grey arrows and brackets. The reactions were carried out in triplicate and representative chromatograms are shown. No reaction products were detected in control reactions lacking reductant or the LPMO (Additional file 1: Fig. S1). Native cello-oligomers, which are present in small amounts, were not identified; they elute in the 2–11 min area and small non-annotated peaks likely belonging to such native products are visible in the 5–11 min sections of the chromatograms

All three WAX substrates (0.2%, w/v) were incubated with 0.2% (w/v) PASC and *Tt*LPMO9E, using gallic acid (GA) as a reductant to drive LPMO activity. A reaction with only PASC was also conducted to generate a standard for cellulose-derived products. The results showed the expected absence of xylan-derived products for the reactions with WAX30 and WAX38 (Fig. 2). On the other hand, the reaction with PASC and WAX22 generated multiple (arabino)xylan-derived products. These peaks were most evident in the 12–25 min region (Fig. 2). Of note, these products were not observed in control reactions without LPMO or reductant (Additional file 1: Fig. S1), which shows that they are indeed the products of LPMO activity. Taken together, the results clearly show that arabinose substitutions inhibit the activity of *Tt*LPMO9E on WAX, but that *Tt*LPMO9E has activity on WAX with lower degrees of substitution.

Next to the effect of substitutions on the WAX–cellulose interaction, variation in the length of the xylan chains could also play a role, with longer chains showing improved adsorption [30], which could increase susceptibility to LPMO action. Based on the data provided by the manufacturer, WAX30 has longer chains than WAX38, while the former was not a better substrate for the LPMO. Therefore, we conclude that debranching, and not chain length, explains why WAX22 is the best substrate for the LPMO, although the effect of molecular weight cannot be completely excluded based on our data.

Due to the complexity of the HPAEC–PAD chromatograms and the lack of suitable analytical standards for arabinoxylan–oligomers, the identity of the LPMO-generated WAX22-derived reaction products could not be determined for the chromatograms. MALDI–TOF MS analysis was not done for these initial experiments with various WAX types. Below, we describe MALDI–TOF MS analyses of other samples of LPMO-treated WAX, which show that mixtures of LPMO-generated WAX-derived reaction products contain multiple species with masses corresponding to native and oxidized xylo-oligomers.

### The activity of *Tt*LPMO9E on WAX22 and WAX38 increases upon debranching with *Um*GH62 α-l-arabinofuranosidase

**Fig. 3:**
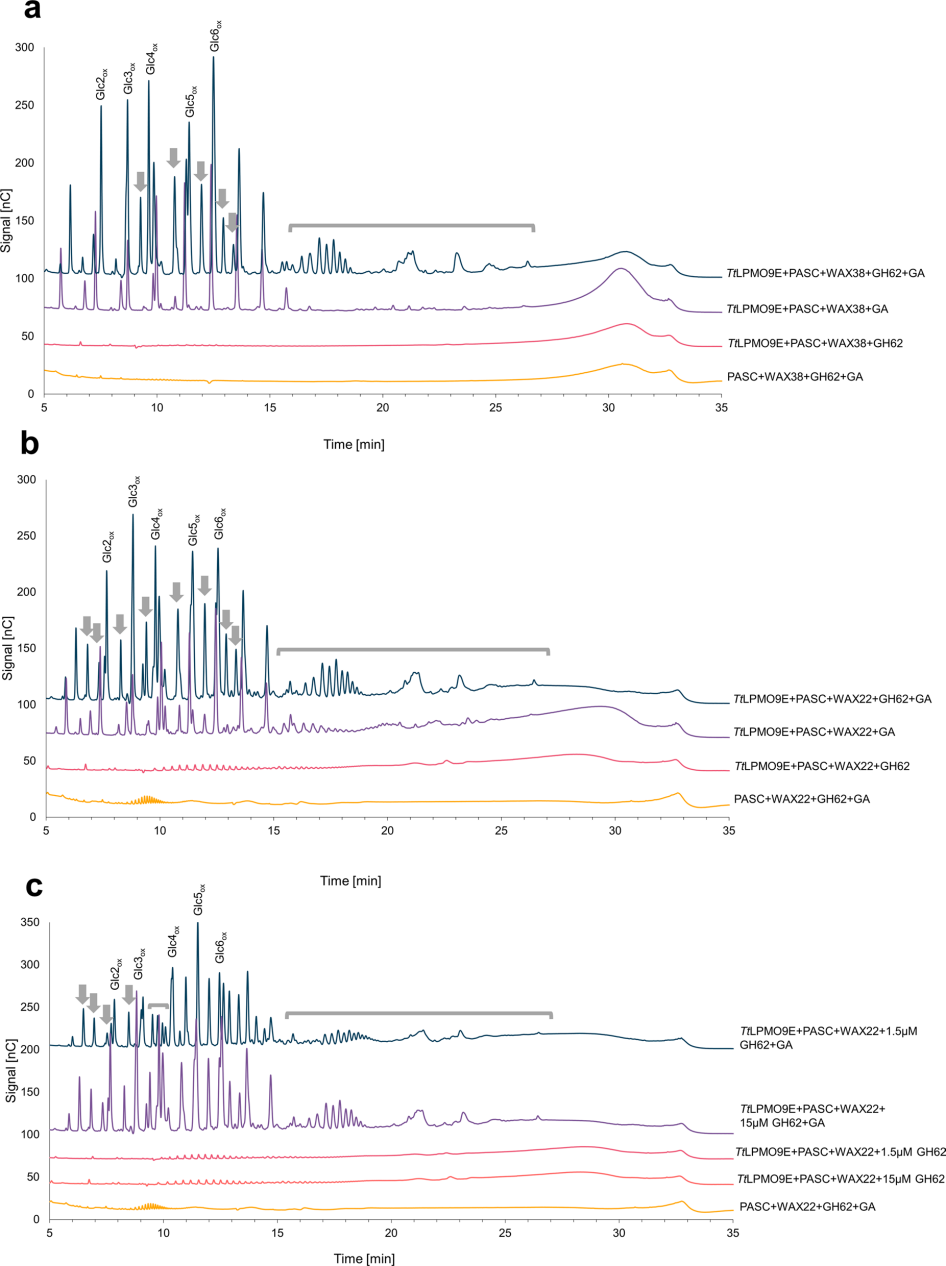
HPAEC–PAD chromatograms showing soluble products generated upon incubation of a mixture of PASC and WAX38 (**a**) or WAX22 (**b**, **c**) with *Tt*LPMO9E and/or *Um*GH62 at pH 6.5 and 40 °C, for 16 h. Panel **c** shows the effect of lowering the dose of *Um*GH62 from 15 µM to 1.5 µM. Oxidized cello-oligosaccharides resulting from PASC degradation are labelled as “GlcX_ox_”, where X indicates the degree of polymerization. Regions that show novel products resulting from LPMO-catalyzed degradation of WAX22 or WAX38 in the presence of *Um*GH62 are indicated by grey arrows and brackets. Control reactions showed that product formation depends on the presence of both the LPMO and the reductant (i.e., a catalytically active LPMO; see panels **a**–**c** and on the presence of cellulose (Additional file 1: Fig. S3, which shows reactions with WAX alone). Control reactions without LPMO contained 0.5 mM CuSO_4_, to account for possible (copper-catalyzed) side reactions

To further test the hypothesis that *Tt*LPMO9E activity on WAX is affected by arabinose substitutions and to investigate whether it was possible to further increase *Tt*LPMO9E activity on WAX, we tested the impact of adding a GH62 arabinofuranosidase to LPMO reactions with PASC and WAX38 or PASC and WAX22. The enzyme used, *Um*GH62, removes α-l-arabinofuranose from singly substituted xylose residues [42] and has a preference for α-1,2 substitutions relative to α-1,3 substitutions according to the enzyme supplier [43]. Figure 3 shows that addition of *Um*GH62 to the reaction mixture enabled *Tt*LPMO9E to degrade WAX38 (Fig. 3a) and increased the activity of the LPMO on WAX22 further (Fig. 3b). The importance of debranching for the *Tt*LPMO9E activity on WAX is evident from the appearance of novel product peaks, as well as peaks with higher intensities in the chromatograms for reactions with *Um*GH62 compared to reactions without *Um*GH62. MALDI–TOF MS analysis of products generated from WAX38 in the absence or presence of the GH62 confirmed the importance of the GH62 for LPMO-catalyzed WAX38 degradation and showed that the product mixture generated in the reaction with both the LPMO and the GH62 contains multiple species with masses corresponding to native and oxidized xylo-oligomers (Additional file 1: Fig. S2). The chromatograms of Fig. 3 show similar, albeit not identical, product patterns for reactions with WAX38 and WAX22; differences appear in the 5–10 min region of the chromatograms (Fig. 3a, b), where the shortest and least substituted products elute. A simple dose–response analysis showed that the amount of *Um*GH62 was not limiting under the conditions used, as a tenfold reduction of the amount of *Um*GH62 in the experiment with PASC + WAX22 only had a limited effect on product formation (Fig. 3c). Control reactions where the LPMO or the reductant were replaced by water did not generate any LPMO-derived products (Fig. 3). Additional control reactions with only WAX38 or WAX22 (Additional file 1: Fig. S3) confirmed that the presence of a cellulosic matrix is essential for the LPMO’s ability to cleave the xylan substrate.

### Debranching with *Um*GH62 arabinofuranosidase and *Bo*GH115A α-(methyl-) glucuronidase improves LPMO activity on spruce and beechwood glucuronoxylans

As the impact of debranching on the activity of *Tt*LPMO9E on WAX was evident, we analyzed if this effect also applied to other types of xylans (Fig. 1), and with other LPMO9s. First, we tested if the previously reported weak activity of *Tt*LPMO9G on spruce arabinoglucuronoxylan (SpAGX) [36] can be boosted by debranching this xylan with *Um*GH62 and/or *Bo*GH115A, a GH115 (methyl)-glucuronidase. The SpAGX substrate used contained 70.6% xylose, 10.7% arabinose and 12.4% MeGlcA (all mol %; [40]). *Bo*GH115A is expected to remove both GlcA and MeGlcA groups (here collectively referred to as (Me)GlcA) with a preference for groups attached to internal xylose residues [41], making it a good candidate for debranching SpAGX.

**Fig. 4:**
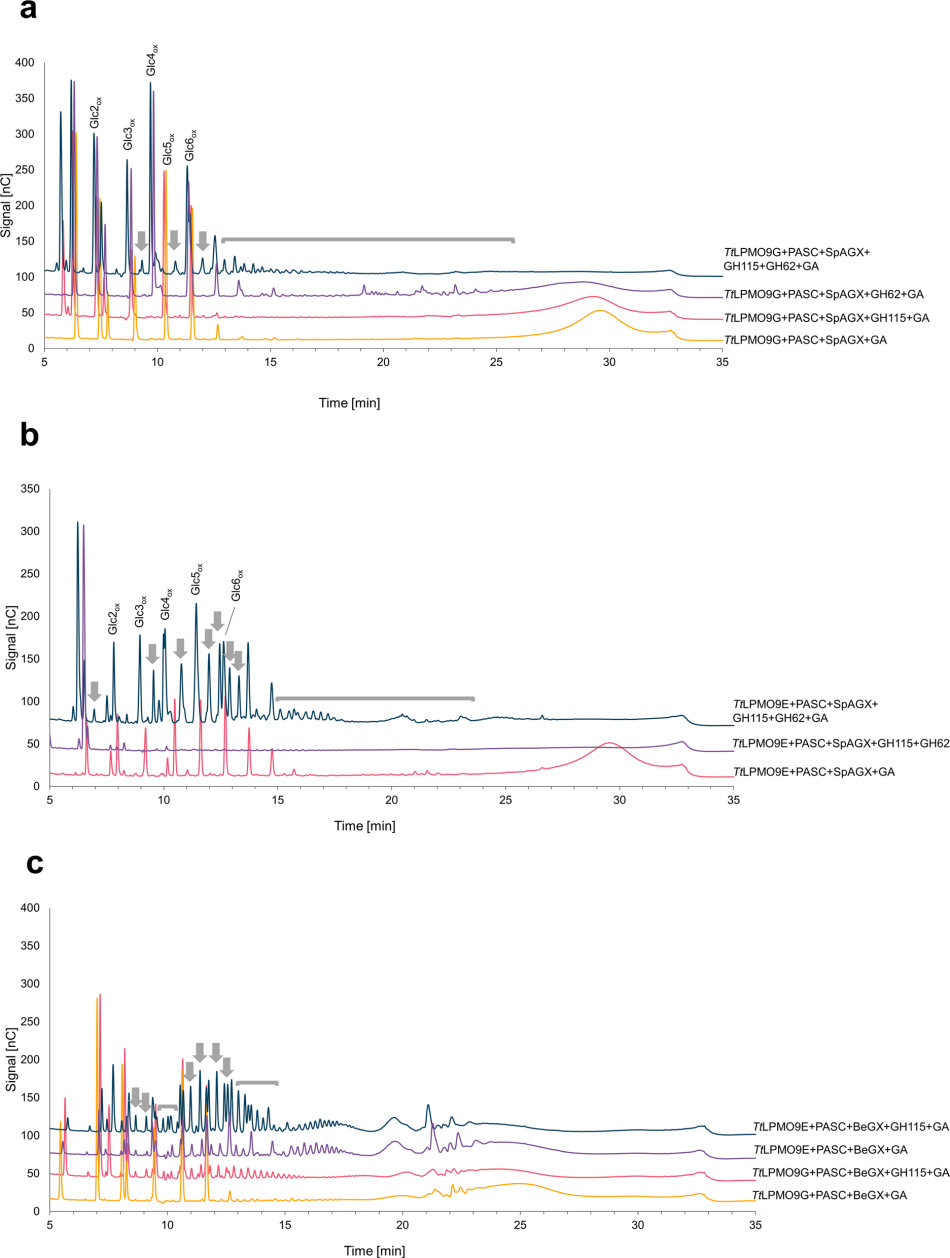
HPAEC–PAD chromatograms showing soluble products generated upon incubation of *Tt*LPMO9E or *Tt*LPMO9G with mixtures of PASC and SpAGX (**a**, **b**) or BeGX (**c**), in the presence or absence of debranching enzymes. Oxidized cello-oligosaccharides resulting from PASC degradation are labelled as “GlcX_ox_”, where X indicates the degree of polymerization. Regions that show novel products resulting from LPMO-catalyzed degradation of SpAGX or BeGX in the presence of the debranching enzymes are indicated by grey arrows and brackets. All reactions were carried out at pH 6.5 and 40 °C, for 16 h. Chromatograms for control reactions without reductant or LPMO did not show oxidized products (Additional file 1: Figs. S4 and S5)

Figure 4a shows that the addition of *Bo*GH115A had minimal effects, if any, on the hardly detectable activity of *Tt*LPMO9G on SpAGX. Minor, barely visible peaks in the 9–14 min elution region suggest that there may be a slight increase in activity due to the addition of *Bo*GH115A. Addition of *Um*GH62 did lead to increased generation of LPMO products, eluting later (16–25 min; Fig. 4a) than the most prominent LPMO products generated from WAX (Figs. 2 and 3). This delayed elution is likely due to the presence of glucuronic acid moieties that add charge to the products, resulting in delayed elution. Most interestingly, the addition of both debranching enzymes led to a clear increase in product generation by *Tt*LPMOG, demonstrating a synergy between the debranching activities of *Bo*GH115A and *Um*GH62 and a need to remove both arabinose and MeGlcA for SpAGX to increase susceptibility to the LPMO. Of note, the potentially glucuronated LPMO products visible in the reaction with *Um*GH62 only were no longer detected in the reaction that also contained *Bo*GH115A. Control reactions without the reductant or without the LPMO (Additional file 1: Fig. S4) confirmed that the products visible in Fig. 4a were indeed a result of LPMO action. Control reactions containing *Bo*GH115A (Additional file 1: Fig. S4) showed a major peak eluting at 7 min, which likely is the released MeGlcA.

In our previous study in which we identified xylan-active LPMO9s from *T. terrestris* [36], *Tt*LPMO9E showed the highest activity on several xylans, but it did not show any activity on SpAGX. Intriguingly, when both *Bo*GH115A and *Um*GH62 were added to a reaction with PASC, SpAGX and *Tt*LPMO9E, SpAGX was readily degraded (Fig. 4b), generating levels of xylan-derived products that were higher than those obtained with *Tt*LPMO9G. The product profiles obtained in the reactions containing *Um*GH62 and *Bo*GH115A differed between *Tt*LPMO9G and *Tt*LPMO9E (Fig. 4a, b), indicating that these two LPMO9s differ in how they tolerate substitutions of the xylan backbone. The lack of dominating cellulose-derived peaks in the chromatogram for the reaction with *Tt*LPMO9E, *Um*GH62 and *Bo*GH115A (Fig. 4b) is noteworthy; this could indicate that debranched SpAGX is as good a substrate for *Tt*LPMO9E as PASC, or even better.

**Fig. 5:**
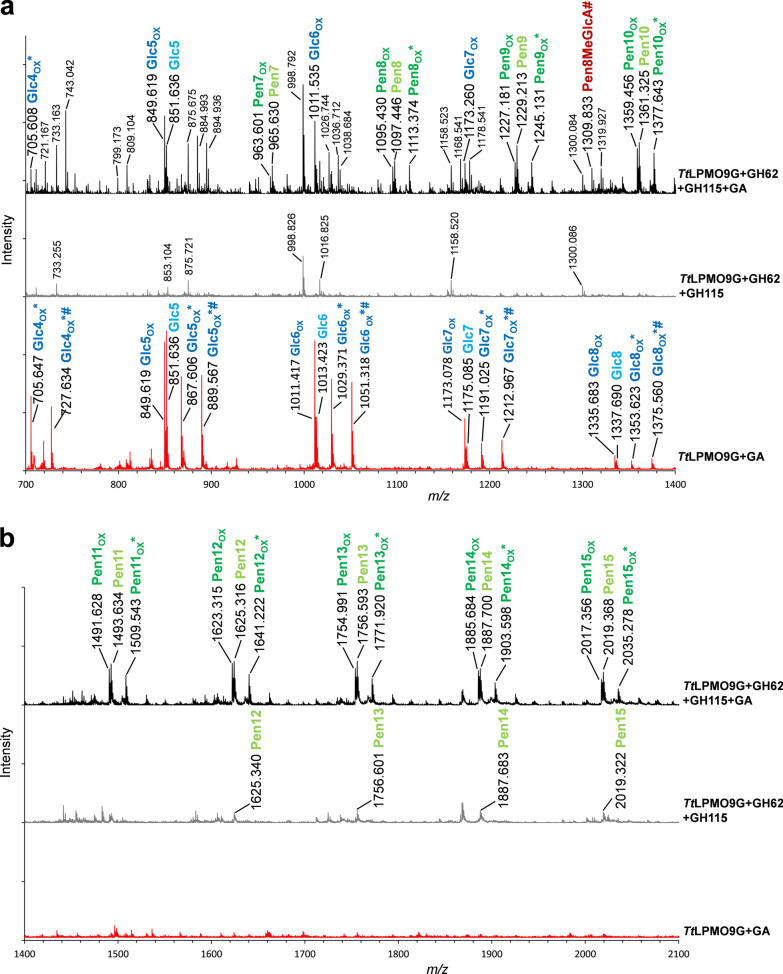
MALDI–TOF MS analysis of products released by *Tt*LPMO9G in reactions with PASC and SpAGX in the presence of *Um*GH62 and *Bo*GH115A. **a** and **b** show the *m/z* regions from 700 to 1400 and from 1400 to 2100, respectively. The top spectra show reactions in the presence of reductant (GA), the middle spectra reductant free controls, and the bottom spectra are control reactions without *Um*GH62 and *Bo*GH115A. Native and oxidized cellulose-derived products (Glc) are labelled blue, and native and oxidized xylan-derived products (Pen) are either labelled green (non-MeGlcA-substituted), or red (MeGlcA-substituted). Light and dark colors represent native and oxidized forms, respectively. “Pen” stands for pentose and indicates xylan-derived products, where the latter may be either linear or substituted with arabinofuranose. All labels refer to single Na-adducts. Hydrated forms of oxidized products are labeled with *, whereas the sodium salts of aldonic or carboxylic acids are labeled with #.

To gain further insight into product formation, soluble compounds generated by *Tt*LPMO9G in the reaction with PASC and SpAGX in the presence of *Um*GH62 and *Bo*GH115A were analyzed by MALDI–TOF MS. The results are displayed in Fig. 5a (700–1400 m/z) and Fig. 5b (1400–2100 m/z). Supporting the results from HPAEC–PAD analysis, the MS spectra showed oxidized reaction products resulting from both cellulose and xylan cleavage. The longest detectable oxidized cellooligomers contained eight glucose units, as is commonly observed due to the limited solubility of longer cellulose fragments. Products originating from xylan degradation were longer, ranging up to at least fifteen pentose monomers. Since the dehydrated masses of both xylopyranose and arabinofuranose units are 132 Da, it is not possible to distinguish if the products are linear (e.g., only xylose) or also include arabinose substitutions. The dominating xylan-derived products are monosodium adducts of native oligomers, oxidized oligomers and hydrated forms of the latter, as well as sodium salts of the hydrated forms (Fig. 5a). These product forms are commonly observed in reactions with C1-oxidizing LPMOs [44, 45]. The MS analysis confirmed that the reductant-free control reaction did not yield oxidized products. In control reactions without *Um*GH62 and *Bo*GH115A, only cellulose-derived products were identified (Fig. 5a, red spectrum). Of note, the mass spectra show several xylan-derived products with masses that corresponded to MeGlcA-substituted (+ 190 Da) xylooligomers. Although MALDI–TOF MS should be considered strictly qualitative, it is interesting to note that the intensities of these product peaks were much weaker than for those belonging to non-MeGlcA-substituted products, which is to be expected considering the presence of *Bo*GH115A in the reaction.

Previously, both *Tt*LPMO9E and, to a much lesser extent, *Tt*LPMO9G have been shown to be active on beechwood glucuronoxylan (BeGX) [36], which is not arabinosylated. Figure 4c shows that addition of *Bo*GH115A to reactions with PASC and BeGX increased the activity of both *Tt*LPMO9s. Interestingly, as also observed above, in the product profile for the reaction with *Tt*LPMO9E and *Bo*GH115A, cellulosic peaks were no longer dominating, again illuminating the high xylanolytic activity of this LPMO. Chromatograms for control reactions without the LPMO or the reductant (Additional file 1: Fig. S5) confirmed that product formation was catalyzed by the LPMO and demonstrated *Bo*GH115A-catalyzed release of (Me)GlcA.

### The type of cellulose affects xylanolytic LPMO9 activity

**Fig. 6:**
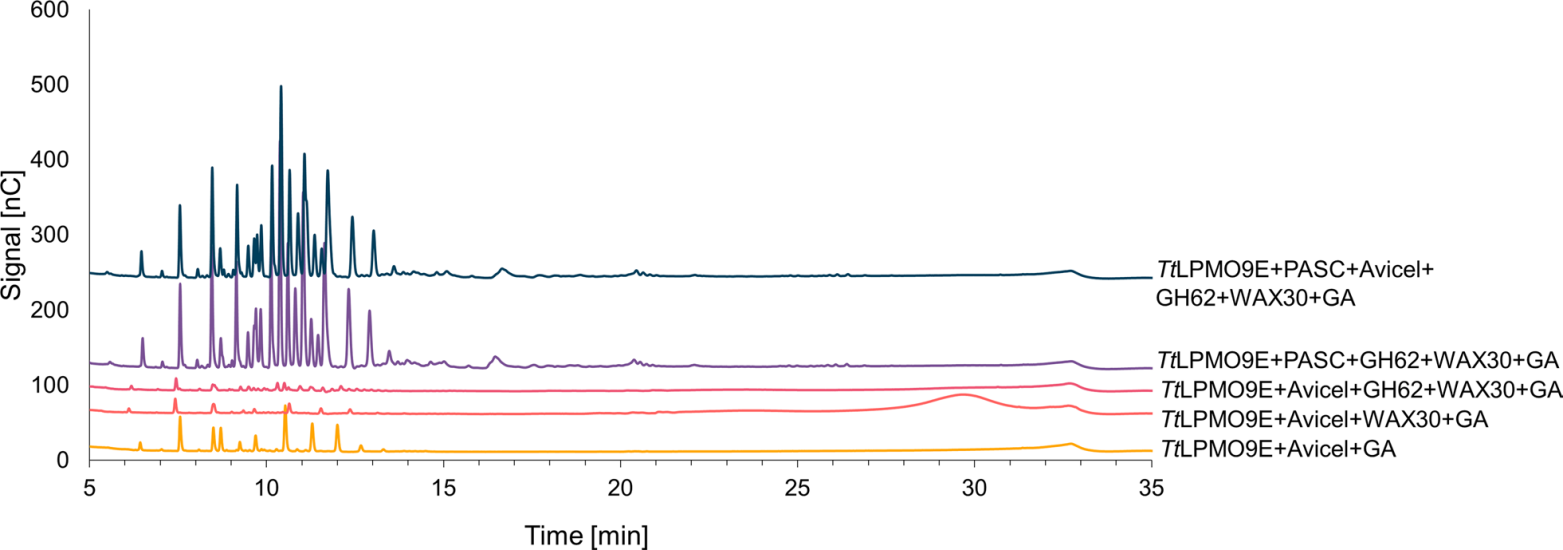
HPAEC–PAD chromatograms showing soluble products generated upon incubation of *Tt*LPMO9E with various mixtures of PASC, Avicel and/or WAX30 in the presence or absence of *Um*GH62. The reactions contained 0.2% (w/v) PASC and/or 0.2% (w/w) Avicel and 0.2% (w/v) WAX30, or 0.4% (w/w) Avicel only, and were performed in 50 mM BisTris–HCl buffer, pH 6.5, at 40 °C, for 16 h. Control reactions without reductant did not generate any oxidized products (Additional file 1: Fig. S6)

Hemicellulose adsorption onto cellulose depends also on the source and related characteristics of the cellulosic material [30, 34]. For instance, Kabel et al. demonstrated in 2007 that xyloglucan adsorbed better to bacterial cellulose compared to Avicel, and hypothesized that this is due to the higher surface area of bacterial cellulose [30]. It has been shown that in PASC, solvent accessible fibril surfaces are more prominent than in Avicel [46]. To get an impression of the impact of different cellulosic substrates, we tested both Avicel and PASC as cellulose matrices in reactions with *Tt*LPMO9E and WAX30, in the absence or presence of *Um*GH62. Reactions with PASC showed clear xylanolytic activity on *Um*GH62-treated WAX30 (Fig. 6), as was to be expected based on the results of similar reactions with WAX22 and WAX38 discussed above (Fig. 3). Unexpectedly, in the reactions with Avicel, the LPMO-catalyzed degradation of WAX30 was almost completely absent (Fig. 6). Addition of PASC to the *Tt*LPMO9E reaction with Avicel, WAX30 and *Um*GH62 restored LPMO activity on xylan, indicating that WAX30 was not irreversibly adsorbed onto Avicel and that WAX30 more readily adsorbs onto PASC compared to Avicel. It is worth noting that Fig. 6 also shows how addition of WAX30 to Avicel hinders LPMO9 activity on Avicel (see lower two chromatograms); this adds to the notion that WAX30 indeed binds to, and coats the Avicel fiber. Control reactions without reductant did not result in any product formation (Additional file 1: Fig. S6).

## Discussion

In the present study, the effect of xylan debranching enzymes on the xylan-degrading activity of family AA9 LPMOs was assessed for the first time. We showed that debranching of xylans can both reveal previously undiscovered xylanolytic activities of LPMOs and significantly boost previously reported activities. Similar improvements in hemicellulose degradation by debranching enzymes have previously been shown for mannanases [47] and xylanases [48–50], but to the best of our knowledge, such studies have not been conducted for LPMO9s prior to the present study.

Although there are studies suggesting that LPMOs may act on free xylans [51–53], convincing activity, i.e., reductant-dependent and with considerable oxidized product formation, has only been detected in reactions that also contain cellulose [8, 14, 16, 36]. This shows that LPMO activity on xylan is dependent on, or at least drastically promoted by, adsorption to cellulose. Improved adsorption is expected since complete debranching of stretches of xylan and/or partial debranching resulting in an even substitution pattern both are known to promote binding of xylan onto cellulose [23, 27, 30–32]. On the other hand, the improved LPMO9 activity upon debranching may result from improved binding of LPMOs to cellulose-adsorbed xylans, as xylan substitutions may cause steric hindrance that prevent productive binding of the LPMO. It has been shown that xylan adsorption onto cellulose tends to leave the substitutions protruding from the xylan surface on the side that is not adsorbed to cellulose [23, 25, 37]. While such protruding groups may prevent productive LPMO binding in some cases, they may also be recognized by LPMO9 that specifically act on certain substituted cellulose–xylan co-polymeric structures. Further studies addressing the conformation of LPMO–substrate complexes are needed to gain insight into these matters. For example, it has recently been shown that sulfate half ester groups on cellulose nanocrystals hamper LPMO10 activity on such sulfated substrates [54], likely due to both steric effects and charge-related repulsion.

Based on our results presented here, and a recent study by Hegnar et al. [16], it is possible to hypothesize that (crystalline) cellulose is not necessarily the preferred substrate for all LPMO9s and that some LPMOs in fact have evolved to act on xylan. For example, we show (Fig. 4) that the soluble products generated in reactions with *Tt*LPMO9E, *Bo*GH115A, *Um*GH62 and mixtures of PASC and BeGX are not dominated by cellulose-derived products, suggesting that cellulose-bound xylan is the preferred substrate (and not PASC). Furthermore, Hegnar et al. [16], quantified oxidized cellulose- and xylan-derived products generated in reactions with LPMO9s and mixtures of PASC and BeGX. They showed that for the most xylan-active LPMO analyzed in the study, *Nc*LPMO9F, the ratio of oxidized xylan-derived products relative to oxidized cellulose-derived products was 3 to 1. One of the other tested enzymes, *Mc*LPMO9H, generated approximately equal amounts of xylan- and cellulose-derived products, whereas cellulose-derived products dominated for the third enzyme investigated in the study, *Nc*LPMO9L. All in all, it is clear that xylan-degradation can be a dominating activity of some LPMO9s, which, however, varies among different enzymes and is dependent on the type of xylan and its interaction with cellulose.

Some LPMO9s are active on multiple types of hemicelluloses. Remarkably, while activity on other hemicelluloses, such as xyloglucan, may be promoted by the presence of cellulose [17], as is the case for xylan, several LPMO9s are active on pure glucomannans and xyloglucans [8, 35, 55]. This variation in cellulose-dependency is likely related to variation in the rigidity and conformational state of hemicelluloses in solution and may also relate to variation in the binding affinities of LPMO9s for these substrates.

It remains to be understood how the substrates used in the work described above relate to true plant cell wall materials, which are more complex co-polymeric structures. It is likely that specific LPMOs have evolved to attack specific co-polymeric substructures in the plant cell wall, such as cellulose-bound xylan, as has been described for an AA14 LPMO [56]. In this context, the differences between the type of cellulose tested (PASC vs. Avicel, Fig. 6) is noteworthy. While we cannot fully explain why the clear boosting effect of *Um*GH62 on WAX30 degradation by *Tt*LPMO9E was not present when Avicel was used as the cellulose matrix instead of PASC, this result clearly shows that the formation of LPMO-susceptible xylan–cellulose complexes also depends on the cellulose. It is also worth noting that the otherwise evident degradation of Avicel by *Tt*LPMO9E was hindered by the presence of WAX30 and *Um*GH62, suggesting that the WAX adsorbs onto Avicel in a way that makes both the xylan and the cellulose inaccessible to cleavage. This observation underpins variation in the co-polymeric structures that may be formed by plant polysaccharides, which translates into variation in their susceptibility to plant biomass degrading enzymes. When PASC was added to the mixture together with Avicel, WAX30 and *Um*GH62, both the xylan- and cellulose-degrading ability of the LPMO were restored, indicating stronger adsorption of WAX30 onto PASC. The biochemical basis for these effects is an interesting starting point for increasing the fundamental understanding of the plant cell wall and thereby putting us in a better position to understand LPMO action. Of note, these findings are also relevant for industrial biomass processing. The various methods that may be used for pretreating lignocellulosic biomass prior to enzymatic digestion will affect the structure and crystallinity of cellulose in different ways, which again may affect the degree and the nature of the association between cellulose and remaining xylan.

The secretomes of lignocellulose-degrading microbes contain a plethora of enzymes that concertedly degrade complex, recalcitrant and co-polymeric substrates. LPMO encoding genes and LPMOs are key components of many such transcriptomes or secretomes, respectively [39, 57, 58]. The present study sheds light on a possible new type of interplay and synergy between members of these secretomes, namely, hemicellulolytic LPMOs and hemicellulose-modifying (debranching) enzymes. Interestingly, a previous analysis of the transcriptomes of *T. terrestris* grown on three different substrates, showed that one of the two GH62 arabinofuranosidase genes is highly upregulated during growth on rice straw (but not during growth on Avicel or beechwood xylan) [39]. Findings like this substantiate the hypothesis that LPMOs might work in concert with xylan- (or other hemicellulose-) debranching enzymes in Nature.

We believe that the connections between LPMOs and hemicellulose debranching enzymes unraveled in the present study may be used to optimize industrial biorefining of lignocellulosic biomass, to produce either fermentable sugars or biomaterials. It is well-known that cellulose-bound recalcitrant xylan inhibits cellulolytic enzyme activity and increases the recalcitrance of lignocellulosic materials [59, 60]. Removal of xylan by LPMOs may improve subsequent saccharification of cellulose and could also be used to produce cleaner cellulose fiber-based biomaterials. It would be interesting to study the effect of debranching enzymes on the impact of LPMOs in lignocellulolytic cocktails. Of note, when doing so, enzyme dosing must be carefully considered as excess debranching may initiate xylan aggregation and/or promote excess cellulase-inhibiting adsorption of xylan onto cellulose. An even deeper molecular-level understanding of how various plant cell wall polymers interact [61, 62] would help in such endeavors.

## Conclusions

A key to solving problems related to the recalcitrance of lignocellulosic biomass and establishing economically feasible biorefineries lies in the optimized use of lytic polysaccharide monooxygenases. So far, the focus has mainly been on AA9 family LPMOs and their role in degrading crystalline cellulose. In the present study, we demonstrated for the first time that xylan debranching enzymes like a GH62 arabinofuranosidase and a GH115 (methyl)glucuronidase increased and even revealed previously hidden xylan-degrading activities of two *Tt*LPMO9s on xylan–PASC mixtures as the cellulose-adsorbed conformation of debranched xylan was likely more accessible to LPMOs. Our findings indicate that in Nature, where a concert of biomass degrading enzymes are present and xylan is found in complex with cellulose, some AA9 LPMOs have likely high capacity to degrade not only cellulose but also xylan. This study illuminates high xylanolytic capabilities of AA9 LPMOs and their functional interplay and likely synergies between other components of complex lignocellulolytic cocktails. Our study paves a way for optimizing the substrate specificities of AA9 LPMOs in future lignocellulolytic cocktails and opens possibilities for exploiting xylanolytic LPMO9s in the production of future biomaterials.

## Methods

### Production and purification of *Tt*LPMO9E and *Tt*LPMO9G

Both *Tt*LPMO9E and *Tt*LPMO9G were heterologously produced, purified and tested for activity as described previously [36]. Shortly, the gene encoding *Tt*LPMO9E, with its natural signal peptide and with no tags attached, was cloned into the pPINK–GAP system [63], and expressed in *Pichia pastoris* PichiaPink strain 4 (Invitrogen, Waltham, MA, United States). The cultivation was carried out for 4 days in 1 L YPD in a 5 L baffled shake flask at 250 rpm and 29 °C. The enzyme was purified from the culture supernatant using ion exchange chromatography as described previously [36]. The *Tt*LPMO9G gene was cloned into the pPICZαA vector and expressed in *P. pastoris* SMD1168H (Invitrogen, Waltham, MA, United States). The expression strain was grown for 5 days in 750 mL BMMY medium in a 5 L baffled shake flask with supplementation of both 1% (v/v) methanol and 1% (w/v) sorbitol at timepoint zero and then every 24 h afterward (our previous work [36] showed that sorbitol supplementation increases LPMO yields with this expression system). The cultivation of this strain was carried out at 200 rpm and 30 °C. The *Tt*LPMO9G gene included a C-terminal His_6_ tag and the protein was purified using gravity flow IMAC. The purities of both enzymes were confirmed using SDS–PAGE and protein concentrations were determined from A_280_ measurements using the respective theoretical extinction coefficients. The pure enzymes were filtered through 0.22 µm Fisherbrand™ sterile PES filters (Thermo Fisher Scientific, Waltham, MA, USA) and stored at 4 °C in 50 mM BisTris-HCl buffer, pH 6.5.

### *Um*GH62 and *Bo*GH115A

The *Ustilago maydis* GH62 α-l-arabinofuranosidase with the product code E-ABFUM was purchased from Megazyme (Bray, Ireland). The high purity recombinant enzyme was supplied as a ~ 200 U/mL suspension in 3.2 M ammonium sulphate and has a pH optimum of 5.0 and temperature optimum of 40 °C. According to Megazyme, one unit of α-l-arabinfuranosidase activity is defined as the amount of enzyme required to release 1 µM of arabinose per minute from 10 mg/mL wheat arabinoxylan in 100 mM sodium acetate buffer at pH 5.0 and 40 °C [43]. The enzyme was stored at 4 °C.

A recombinantly produced GH115 α-(methyl)-glucuronidase from *Bacteroides ovatus* with product code CZ0311 was purchased from NZYTech (Lisbon, Portugal). *Bo*GH115A was supplied at 1 mg/mL in 35 mM NaHepes buffer, pH 7.5, 750 mM NaCl, 200 mM imidazol, 3.5 mM CaCl_2_ and 25% (v/v) glycerol. The ≥ 90% pure *Bo*GH115A was reported to have a pH optimum of 7.0 and a temperature optimum of 37 °C. The enzyme was stored at − 20 °C. For both enzymes, protein concentrations were analyzed using A_280_ measurements and the theoretical extinction coefficients.

### Substrates

PASC was produced from Avicel PH-101 according to Wood et al. [64]. Avicel PH-101 (from cotton linters) was from Sigma-Aldrich, beechwood glucuronoxylan (product code BI3856) was from Apollo Scientific (Cheshire, UK), low viscosity wheat arabinoxylan (38:62 Ara:Xyl, product code P-WAXYL), enzymatically debranched wheat arabinoxylan (30:70 Ara:Xyl, product code P-EDWAX30) and acid debranched wheat arabinoxylan (22:78 Ara:Xyl, product code P-ADWAX22) were purchased from Megazyme (Bray, Ireland). All WAX substrates were reported to be > 94% pure on a dry weight basis (info for BeGX was not available). AGX from spruce was produced in-house as described in [40]. All substrates were dissolved to 1% (w/v) in water (the Megazyme substrates according to the producer’s manual), except for Avicel, which was suspended at 1% (w/w) in water.

### Enzymatic reactions

All reactions, except for those with Avicel instead of PASC, were set up in triplicates with 100 µL reaction volume in 1.5 mL Eppendorf tubes and incubated in orbital thermomixers set at 1000 rpm. Reactions with Avicel were set up with a 500 µL final volume, in duplicates, in 2 mL Eppendorf tubes, using head-to-tail mixing at 60 rpm. All reactions were carried out in 50 mM BisTris-HCl buffer at pH 6.5 and at 40 °C, which are conditions that favor LPMO activity, while being suboptimal for the *Um*GH62 and *Bo*GH115A. According to Megazyme, *Um*GH62 displays at least 40% of its maximum activity at pH 6.5 (respective data not available for *Bo*GH115A). All reactions contained 1 µM LPMO (incubated with 0.5 mM CuSO_4_ on ice, minimum 30 min before starting the reactions), 0.2% (w/v) hemicellulose and 0.2% cellulose (w/v for PASC, w/w for Avicel) for double substrate reactions, or 0.4% (for Avicel w/w, for others w/v) polysaccharide for single substrate reactions. Where applicable, the final *Bo*GH115A concentration was 0.1 µM. The final *Um*GH62 concentration was 15 µM (approximately 0.45 U), except for the dose–response experiment in which also a tenfold lower concentration (1.5 µM, approximately 0.045 U) was tested. In reactions with LPMO and reductant, the final component added to the reaction mixture was gallic acid (GA) (at t = 0) to initiate the reactions (1 mM final concentration). The control reactions without LPMOs, also contained 0.5 mM CuSO_4_ to account for possible (copper-catalyzed) side-reactions. *Um*GH62 and/or *Bo*GH115A were added shortly after the LPMO but before GA addition. All reactions were incubated for 16 h and stopped by incubating for 15 min at 99 °C, followed by filtering through 0.45 µm filter plates (Millipore, Darmstadt, Germany). In reactions containing *Um*GH62, including control reactions without the LPMO, boiling led to visible precipitation, which was likely due to the reduced solubility of xylan that results from removing arabinose substitutions [31–33]. The filtrates were stored at − 20 °C prior to product analysis.

### Product analysis by HPAEC–PAD

A Dionex ICS5000 system with a 3 × 250 mm Carbopac PA200 analytical and a 3 × 50 mm Carbopac PA200 guard column (Dionex, Sunnywale, CA, USA) was used for chromatographic analysis of soluble native and oxidized cello- and xylooligosaccharides. Prior to chromatographic analysis, the filtered samples were diluted fourfold in MilliQ water. 10 µL of sample was injected to the column. All replicates were analyzed using HPAEC–PAD and in all cases the chromatograms of the replicates looked highly similar. Thus, for each reaction only one chromatogram is presented in the figures. The gradient and other conditions used for the HPAEC–PAD analysis have been described previously [36].

### Product analysis by MALDI–TOF MS

Masses of generated oxidized and non-oxidized products were analyzed by matrix-assisted laser desorption/ionization time-of-flight mass spectrometry (MALDI–TOF MS). The used instrument was from Ultraflex (Bruker Daltonics, Billerica, MA, USA) and was equipped with a 337-nm laser in positive reflector mode, as described previously [13]. To optimize product detection and to saturate the samples with sodium (which reduces spectrum complexity), the samples were diluted four times in 5 mM BisTris-HCl, pH 6.0, containing 15 mM NaCl (pH 6.0), as described previously [36], after which 1.5 µL of sample was mixed with 1.5 µL MALDI matrix solution (10 mg/mL 2,5-dihydroxybenzoic acid in 30% (v/v) acetonitrile and 0.1% (v/v) trifluoroacetic acid) on an MTP BigAnchor 384 BC ground steel target plate (Bruker Daltonics). Samples were dried in ambient air. 85% laser intensity was used, with 1000 shots. The spectra were collected using flexControl and analyzed using mMass [65].

## Supplementary Information

**Additional file 1: Figure S1. **HPAEC–PAD chromatograms for control reactions related to the assessment of *Tt*LPMO9E activity on differently substituted WAX. **Figure S2.** MALDI–TOF MS spectra of LPMO derived products. **Figure S3.** HPAEC–PAD chromatograms for reactions related to the assessment of the combined activity of *Tt*LPMO9E and *Um*GH62 on WAX22 and WAX38 without PASC as a cellulose matrix. **Figure S4.** HPAEC–PAD chromatograms for control reactions related to the assessment of LPMO9 activity on a mixture of PASC and SpAGX in the presence or absence of a *Um*GH62 and/or a *Bo*GH115A. **Figure S5.** HPAEC–PAD chromatograms for control reactions related to the assessment of LPMO9 activity on a mixture of PASC and BeGX in the presence or absence of *Bo*GH115A. **Figure S6.** HPAEC–PAD chromatograms for control reactions related to the assessment of *Tt*LPMO9E activity on WAX30 in the presence of PASC and/or Avicel and in the absence or presence of *Um*GH62.

## Data Availability

All relevant data have been included in the article and the supporting information.

## Abbreviations

LPMO: Lytic polysaccharide monooxygenase
AA9: Auxiliary activity family 9
LPMO9: Lytic polysaccharide monooxygenase from auxiliary activity family 9
PASC: Phosphoric acid swollen cellulose
RAC: Regenerated amorphous cellulose
WAX: Wheat arabinoxylan
GA: Gallic acid
SpAGX: Spruce arabinoglucuronoxylan
BeGX: Beechwood glucuronoxylan
GH: Glycoside hyrdolase
HPAEC-PAD: High-performance anion exchange chromatography with pulsed amperometric detection
MALDI-TOF MS: Matrix-assisted laser desorption/ionization time-of-flight mass spectrometry
Ara: Arabinose
Xyl: Xylose
(Me)GlcA: (Methyl)glucuronic acid

## References

[1] Vaaje-Kolstad G, Westereng B, Horn SJ, Liu Z, Zhai H, Sørlie M (2010). An oxidative enzyme boosting the enzymatic conversion of recalcitrant polysaccharides. Science.

[2] Quinlan RJ, Sweeney MD, Lo Leggio L, Otten H, Poulsen JCN, Johansen KS (2011). Insights into the oxidative degradation of cellulose by a copper metalloenzyme that exploits biomass components. Proc Natl Acad Sci U S A.

[3] Bissaro B, Røhr ÅK, Müller G, Chylenski P, Skaugen M, Forsberg Z (2017). Oxidative cleavage of polysaccharides by monocopper enzymes depends on H_2_O_2_. Nat Chem Biol.

[4] Chylenski P, Bissaro B, Sørlie M, Røhr ÅK, Várnai A, Horn SJ (2019). Lytic polysaccharide monooxygenases in enzymatic processing of lignocellulosic biomass. ACS Catal.

[5] Drula E, Garron ML, Dogan S, Lombard V, Henrissat B, Terrapon N (2022). The carbohydrate-active enzyme database: functions and literature. Nucleic Acids Res.

[6] Bey M, Zhou S, Poidevin L, Henrissat B, Coutinho PM, Berrin J-G (2013). Cello-oligosaccharide oxidation reveals differences between two lytic polysaccharide monooxygenases (family GH61) from *Podospora anserina*. Appl Environ Microbiol.

[7] Chen C, Chen J, Geng Z, Wang M, Liu N, Li D (2018). Regioselectivity of oxidation by a polysaccharide monooxygenase from *Chaetomium thermophilum*. Biotechnol Biofuels.

[8] Hüttner S, Várnai A, Petrovic D, Bach CX, Kim Anh DT, Thanh VN (2019). Specific xylan activity revealed for AA9 Lytic polysaccharide monooxygenases of the thermophilic fungus *Malbranchea cinnamomea* by functional characterization. Appl Environ Microbiol.

[9] Vu VV, Beeson WT, Phillips CM, Cate JHD, Marletta MA (2014). Determinants of regioselective hydroxylation in the fungal polysaccharide monooxygenases. J Am Chem Soc.

[10] Sun P, Laurent CVFP, Boerkamp VJP, van Erven G, Ludwig R, van Berkel WJH (2022). Regioselective C4 and C6 double oxidation of cellulose by lytic polysaccharide monooxygenases. Chemsuschem.

[11] Hemsworth GR, Johnston EM, Davies GJ, Walton PH (2015). Lytic polysaccharide monooxygenases in biomass conversion. Trends Biotechnol.

[12] Østby H, Hansen LD, Horn SJ, Eijsink VGH, Várnai A (2020). Enzymatic processing of lignocellulosic biomass: principles, recent advances and perspectives. J Ind Microbiol Biotechnol.

[13] Agger JW, Isaksen T, Várnai A, Vidal-melgosa S, Willats WGT, Ludwig R (2014). Discovery of LPMO activity on hemicelluloses shows the importance of oxidative processes in plant cell wall degradation. Proc Natl Acad Sci U S A.

[14] Frommhagen M, Sforza S, Westphal AH, Visser J, Hinz SWA, Koetsier MJ (2015). Discovery of the combined oxidative cleavage of plant xylan and cellulose by a new fungal polysaccharide monooxygenase. Biotechnol Biofuels.

[15] Bennati-Granier C, Garajova S, Champion C, Grisel S, Haon M, Zhou S (2015). Substrate specificity and regioselectivity of fungal AA9 lytic polysaccharide monooxygenases secreted by *Podospora anserina*. Biotechnol Biofuels.

[16] Hegnar OA, Østby H, Petrović DM, Olsson L, Várnai A, Eijsink VGH (2021). Quantifying oxidation of cellulose-associated glucuronoxylan by two lytic polysaccharide monooxygenases from *Neurospora crassa*. Appl Environ Microbiol.

[17] Kojima Y, Várnai A, Ishida T, Sunagawa N, Petrovic DM, Igarashi K (2016). A lytic polysaccharide monooxygenase with broad xyloglucan specificity from the brown-rot fungus *Gloeophyllum trabeum* and its action on cellulose-xyloglucan complexes. Appl Environ Microbiol.

[18] Albersheim P, Darvill A, Roberts K, Sederoff R, Staehelin A (2010). Plant cell walls.

[19] Scheller HV, Ulvskov P (2010). Hemicelluloses. Annu Rev Plant Biol.

[20] Gellerstedt G, Ek M, Henriksson G (2009). Wood chemistry and biotechnology.

[21] Chong SL, Virkki L, Maaheimo H, Juvonen M, Derba-Maceluch M, Koutaniemi S (2014). O-Acetylation of glucuronoxylan in *Arabidopsis thaliana* wild type and its change in xylan biosynthesis mutants. Glycobiology.

[22] Bromley JR, Busse-Wicher M, Tryfona T, Mortimer JC, Zhang Z, Brown DM (2013). GUX1 and GUX2 glucuronyltransferases decorate distinct domains of glucuronoxylan with different substitution patterns. Plant J.

[23] Busse-Wicher M, Gomes TCFF, Tryfona T, Nikolovski N, Stott K, Grantham NJ (2014). The pattern of xylan acetylation suggests xylan may interact with cellulose microfibrils as a twofold helical screw in the secondary plant cell wall of *Arabidopsis thaliana*. Plant J.

[24] Martínez-Abad A, Giummarella N, Lawoko M, Vilaplana F (2018). Differences in extractability under subcritical water reveal interconnected hemicellulose and lignin recalcitrance in birch hardwoods. Green Chem.

[25] Busse-Wicher M, Li A, Silveira RL, Pereira CS, Tryfona T, Gomes TCF (2016). Evolution of xylan substitution patterns in Gymnosperms and Angiosperms: implications for xylan interaction with cellulose. Plant Physiol.

[26] Martínez-Abad A, Berglund J, Toriz G, Gatenholm P, Henriksson G, Lindström M (2017). Regular motifs in xylan modulate molecular flexibility and interactions with cellulose surfaces. Plant Physiol.

[27] Grantham NJ, Wurman-Rodrich J, Terrett OM, Lyczakowski JJ, Stott K, Iuga D (2017). An even pattern of xylan substitution is critical for interaction with cellulose in plant cell walls. Nat Plants.

[28] Terrett OM, Lyczakowski JJ, Yu L, Iuga D, Franks WT, Brown SP (2019). Molecular architecture of softwood revealed by solid-state NMR. Nat Commun.

[29] Martínez-Abad A, Jiménez-Quero A, Wohlert J, Vilaplana F (2020). Influence of the molecular motifs of mannan and xylan populations on their recalcitrance and organization in spruce softwoods. Green Chem.

[30] Kabel MA, van den Borne H, Vincken JPP, Voragen AGJJ, Schols HA (2007). Structural differences of xylans affect their interaction with cellulose. Carbohydr Polym.

[31] Köhnke T, Östlund Å, Brelid H (2011). Adsorption of arabinoxylan on cellulosic surfaces: influence of degree of substitution and substitution pattern on adsorption characteristics. Biomacromol.

[32] Bosmans TJ, Stépán AM, Toriz G, Renneckar S, Karabulut E, Wågberg L (2014). Assembly of debranched xylan from solution and on nanocellulosic surfaces. Biomacromol.

[33] Shrestha UR, Smith S, Pingali SV, Yang H, Zahran M, Breunig L (2019). Arabinose substitution effect on xylan rigidity and self-aggregation. Cellulose.

[34] Gu J, Catchmark JM (2013). The impact of cellulose structure on binding interactions with hemicellulose and pectin. Cellulose.

[35] Sun P, de Munnik M, van Berkel WJH, Kabel MA (2022). Extending the diversity of *Myceliophthora thermophila* LPMOs: Two different xyloglucan cleavage profiles. Carbohydr Polym.

[36] Tõlgo M, Hegnar OA, Østby H, Várnai A, Vilaplana F, Eijsink VGH (2022). Comparison of six lytic polysaccharide monooxygenases from *Thermothielavioides terrestris* shows that functional variation underlies the multiplicity of LPMO genes in filamentous fungi. Appl Environ Microbiol.

[37] Simmons TJ, Mortimer JC, Bernardinelli OD, Pöppler AC, Brown SP, DeAzevedo ER (2016). Folding of xylan onto cellulose fibrils in plant cell walls revealed by solid-state NMR. Nat Commun.

[38] Berka RM, Grigoriev IV, Otillar R, Salamov A, Grimwood J, Reid I (2011). Comparative genomic analysis of the thermophilic biomass-degrading fungi *Myceliophthora thermophila* and *Thielavia terrestris*. Nat Biotechnol.

[39] Tõlgo M, Hüttner S, Rugbjerg P, Thuy NT, Thanh VN, Larsbrink J (2021). Genomic and transcriptomic analysis of the thermophilic lignocellulose-degrading fungus *Thielavia terrestris* LPH172. Biotechnol Biofuels.

[40] Berglund J, Mikkelsen D, Flanagan BM, Dhital S, Gaunitz S, Henriksson G (2020). Wood hemicelluloses exert distinct biomechanical contributions to cellulose fibrillar networks. Nat Commun.

[41] Rogowski A, Baslé A, Farinas CS, Solovyova A, Mortimer JC, Dupree P (2014). Evidence that GH115 α-glucuronidase activity, which is required to degrade plant biomass, is dependent on conformational flexibility. J Biol Chem.

[42] Siguier B, Haon M, Nahoum V, Marcellin M, Burlet-Schiltz O, Coutinho PM (2014). First structural insights into α-l-Arabinofuranosidases from the two GH62 glycoside hydrolase subfamilies. J Biol Chem.

[43] Megazyme. α-L-Arabinofuranosidase *(Ustilago maydis)* [Internet]. https://www.megazyme.com/alpha-l-arabinofuranosidase-ustilago-maydis?sSearch=GH62. Accessed 24 Jul 2022.

[44] Westereng B, Arntzen M, Agger JW, Vaaje-Kolstad G, Eijsink VGH (2017). Analyzing activities of LPMO by liquid chromatography and mass spectrometry. Protein-carbohydrate Interact Methods Protoc.

[45] Calderaro F, Bevers LE, van den Berg MA (2021). Oxidative power: tools for assessing LPMO activity on cellulose. Biomolecules.

[46] Zhang X, Qu T, Mosier NS, Han L, Xiao W (2018). Cellulose modification by recyclable swelling solvents. Biotechnol Biofuels.

[47] Arnling Bååth J, Martínez-Abad A, Berglund J, Larsbrink J, Vilaplana F, Olsson L (2018). Mannanase hydrolysis of spruce galactoglucomannan focusing on the influence of acetylation on enzymatic mannan degradation. Biotechnol Biofuels.

[48] Li X, Kouzounis D, Kabel MA, de Vries RP (2022). GH10 and GH11 endoxylanases in *Penicillium subrubescens*: comparative characterization and synergy with GH51, GH54, GH62 α-L-arabinofuranosidases from the same fungus. N Biotechnol.

[49] McKee LS, Sunner H, Anasontzis GE, Toriz G, Gatenholm P, Bulone V (2016). A GH115 α-glucuronidase from *Schizophyllum commune* contributes to the synergistic enzymatic deconstruction of softwood glucuronoarabinoxylan. Biotechnol Biofuels.

[50] Kmezik C, Bonzom C, Olsson L, Mazurkewich S, Larsbrink J (2020). Multimodular fused acetyl-feruloyl esterases from soil and gut *Bacteroidetes* improve xylanase depolymerization of recalcitrant biomass. Biotechnol Biofuels.

[51] Basotra N, Dhiman SS, Agrawal D, Sani RK, Tsang A, Chadha BS (2019). Characterization of a novel lytic polysaccharide monooxygenase from *Malbranchea cinnamomea* exhibiting dual catalytic behavior. Carbohydr Res.

[52] Chorozian K, Karnaouri A, Karantonis A, Souli M, Topakas E (2022). Characterization of a dual cellulolytic/xylanolytic AA9 lytic polysaccharide monooxygenase from *Thermothelomyces thermophilus* and its utilization toward nanocellulose production in a multi-step bioprocess. ACS Sustain Chem Eng.

[53] Simmons TJ, Frandsen KEH, Ciano L, Tryfona T, Lenfant N, Poulsen JC (2017). Structural and electronic determinants of lytic polysaccharide monooxygenase reactivity on polysaccharide substrates. Nat Commun.

[54] Solhi L, Li J, Li J, Heyns NMI, Brumer H (2022). Oxidative enzyme activation of cellulose substrates for surface modification. Green Chem.

[55] Monclaro AV, Petrović DM, Alves GSC, Costa MMC, Midorikawa GEO, Miller RNG (2020). Characterization of two family AA9 LPMOs from *Aspergillus tamarii* with distinct activities on xyloglucan reveals structural differences linked to cleavage specificity. PLoS ONE.

[56] Couturier M, Ladevèze S, Sulzenbacher G, Ciano L, Fanuel M, Moreau C (2018). Lytic xylan oxidases from wood-decay fungi unlock biomass degradation. Nat Chem Biol.

[57] Hüttner S, Nguyen TT, Granchi Z, Chin-A-Woeng T, Ahrén D, Larsbrink J (2017). Combined genome and transcriptome sequencing to investigate the plant cell wall degrading enzyme system in the thermophilic fungus *Malbranchea cinnamomea*. Biotechnol Biofuels.

[58] Arntzen M, Bengtsson O, Várnai A, Delogu F, Mathiesen G, Eijsink VGH (2020). Quantitative comparison of the biomass-degrading enzyme repertoires of five filamentous fungi. Sci Rep.

[59] Várnai A, Huikko L, Pere J, Siika-aho M, Viikari L (2011). Synergistic action of xylanase and mannanase improves the total hydrolysis of softwood. Bioresour Technol.

[60] Hu J, Arantes V, Saddler JN (2011). The enhancement of enzymatic hydrolysis of lignocellulosic substrates by the addition of accessory enzymes such as xylanase: is it an additive or synergistic effect?. Biotechnol Biofuels.

[61] Carpita NC, McCann MC (2020). Redesigning plant cell walls for the biomass-based bioeconomy. J Biol Chem.

[62] Busse-Wicher M, Grantham NJ, Lyczakowski JJ, Nikolovski N, Dupree P (2016). Xylan decoration patterns and the plant secondary cell wall molecular architecture. Biochem Soc Trans.

[63] Várnai A, Tang C, Bengtsson O, Atterton A, Mathiesen G, Eijsink VGH (2014). Expression of endoglucanases in *Pichia pastoris* under control of the GAP promoter. Microb Cell Fact.

[64] Wood TM. Preparation of crystalline, amorphous, and dyed cellulase substrates. In: Methods in enzymology. Academic Press; 1988. p. 19–25. 10.1016/0076-6879(88)60103-0

[65] Niedermeyer THJ, Strohalm M (2012). mMass as a software tool for the annotation of cyclic peptide tandem mass spectra. PLoS ONE.

