# Supplementary material for: Enzymatic debranching is a key determinant of the xylan-degrading activity of family AA9 lytic polysaccharide monooxygenases
Source: Biotechnol Biofuels Bioprod. 2023 Jan 5;16:2. doi: 10.1186/s13068-022-02255-2 (PMC9814446; doi:10.1186/s13068-022-02255-2)
Supplement: Supplementary file 1 — Additional file 1: Figure S1. HPAEC–PAD chromatograms for control reactions related to the assessment of TtLPMO9E activity on differently substituted WAX. Figure S2. MALDI–TOF MS spectra of LPMO derived products. Figure S3. HPAEC–PAD chromatograms for reactions related to the assessment of the combined activity of TtLPMO9E and UmGH62 on WAX22 and WAX38 without PASC as a cellulose matrix. Figure S4. HPAEC–PAD chromatograms for control reactions related to the assessment of LPMO9 activity on a mixture of PASC and SpAGX in the presence or absence of a UmGH62 and/or a BoGH115A. Figure S5. HPAEC–PAD chromatograms for control reactions related to the assessment of LPMO9 activity on a mixture of PASC and BeGX in the presence or absence of BoGH115A. Figure S6. HPAEC–PAD chromatograms for control reactions related to the assessment of TtLPMO9E activity on WAX30 in the presence of PASC and/or Avicel and in the absence or presence of UmGH62. [file 13068_2022_2255_MOESM1_ESM.docx]

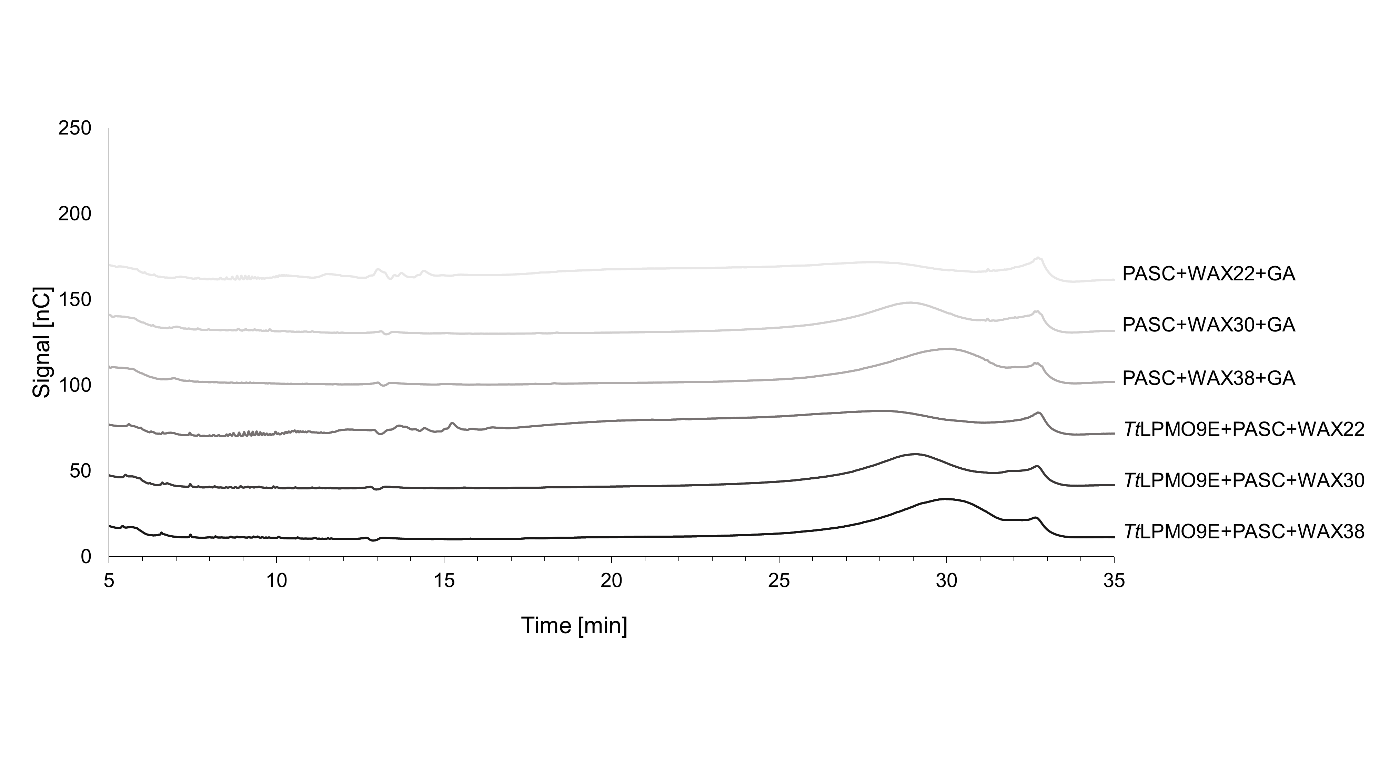


**Figure S1. HPAEC-PAD chromatograms for control reactions related to the assessment of *Tt*LPMO9E activity on differently substituted WAX.** In these control reactions, related to Figure 2 in the main manuscript, a combination of PASC and differently substituted WAX was incubated for 16 h at pH 6.5 and 40 °C in the absence of either the LPMO or the reductant gallic acid (GA), which were replaced by water. The control reactions without LPMO contained 0.5 μM CuSO_4_, to account for possible (copper-catalyzed) side reactions.


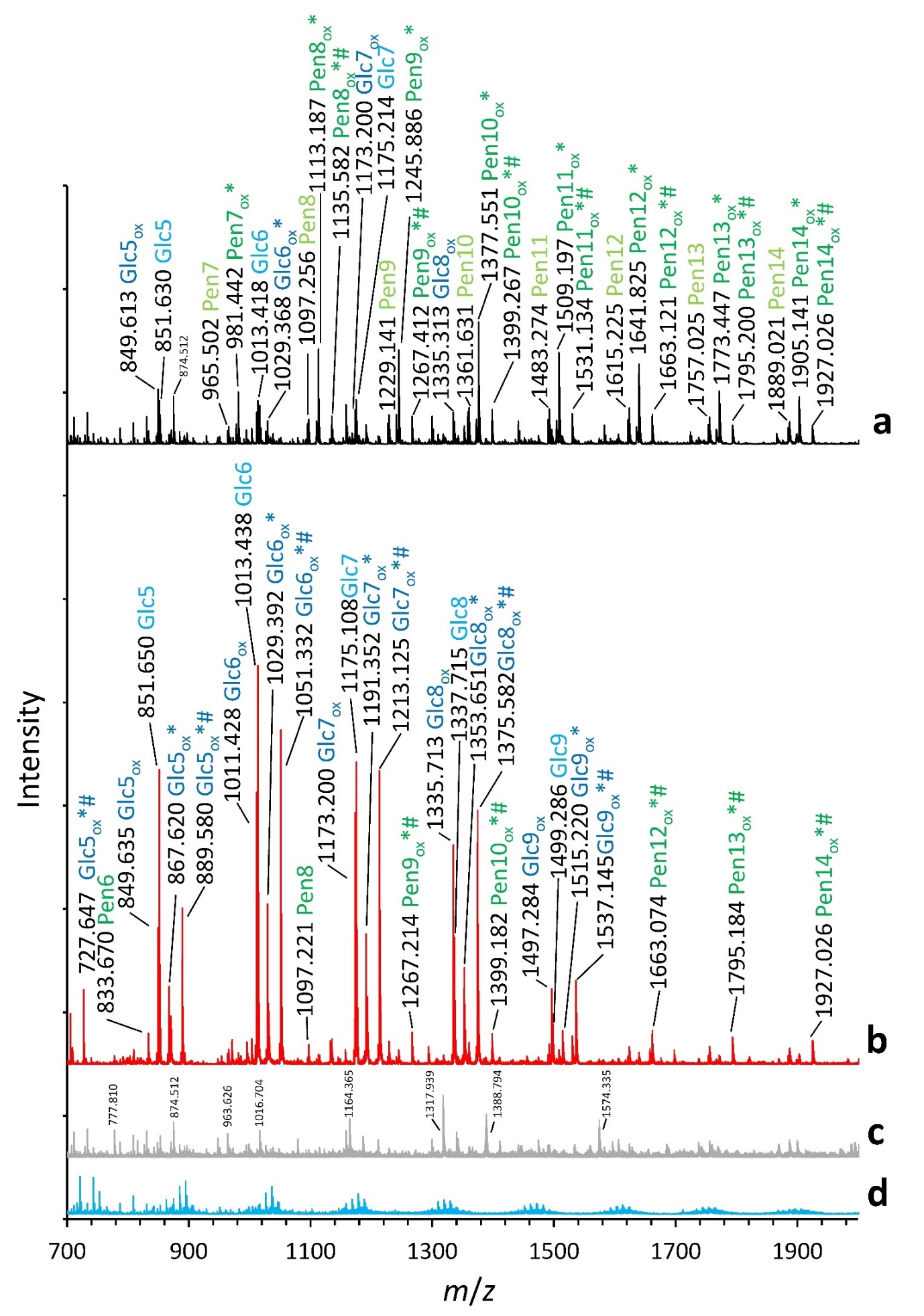


**Fig S2. MALDI-TOF MS spectra of LPMO derived products** generated in reactions with **a**) PASC+WAX38+*Tt*LPMO9E+*Um*GH62+GA, **b**) PASC+WAX38+*Tt*LPMO9E+GA and **c)** PASC+WAX38+*Tt*LPMO9E+*Um*GH62 (no reductant). Spectrum **d)** shows the substrate control without LPMO, containing PASC+WAX38+*Um*GH62+GA. Reactions were performed with 1 μM LPMO (replaced with water for **d**), 0.2 % PASC (w/v%), 0.2 % WAX38 (w/v%), 15 μM GH62 (replaced with water for **b**). All reactions were performed in the presence of 0.5 μM CuSO_4_ in 50 mM BisTris-HCl pH 6.5 at 40 °C for 24 h. Light blue products represent native cello-oligomers, dark blue oxidized cello-oligomers, light green native pentose oligomers and dark green oxidized pentose oligomers. Sodium adducts are indicated with #, and hydrated forms of oxidized products are indicated with *. Note that further product identification of the WAX-derived products is not possible because of the identical masses of arabinofuranose and xylopyranose.


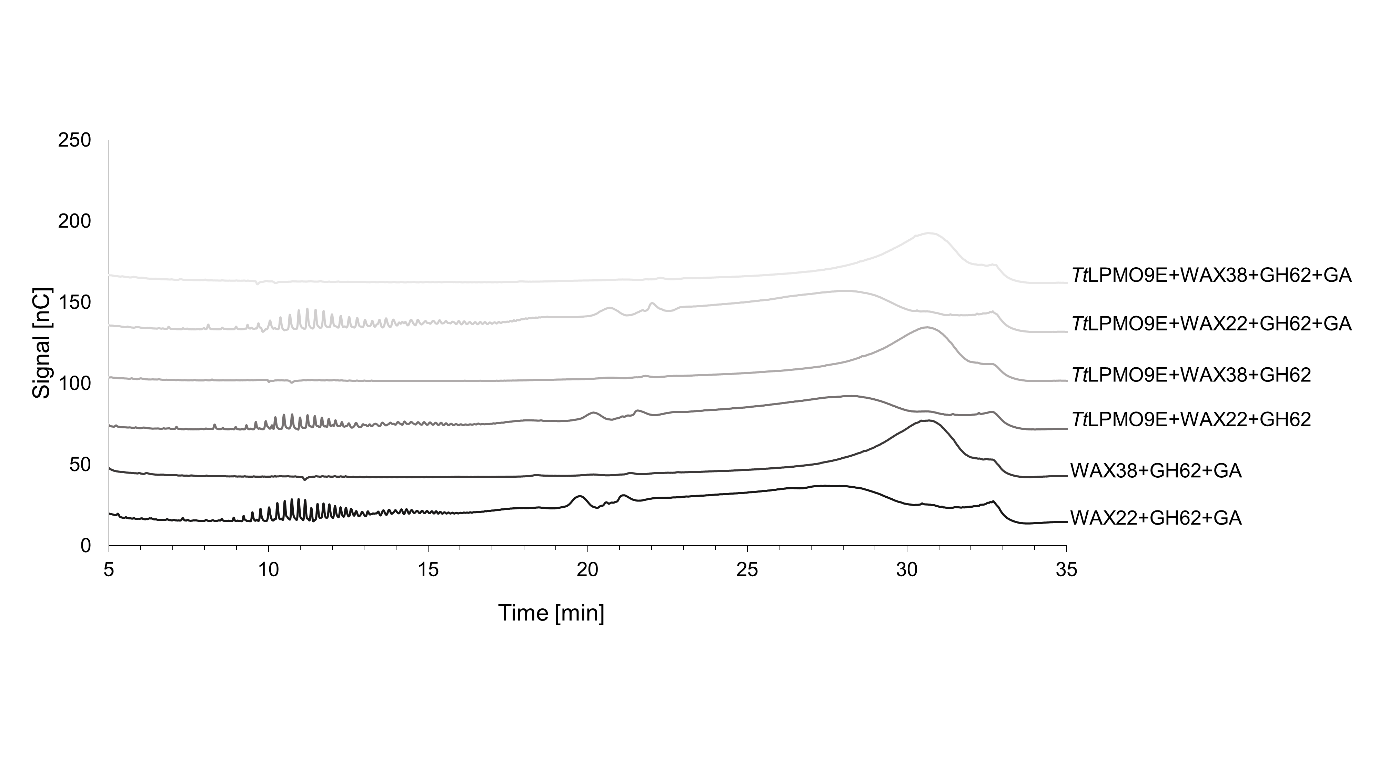


**Figure S3.** **HPAEC-PAD chromatograms for reactions related to the assessment of the combined activity of *Tt*LPMO9E and *Um*GH62 on WAX22 and WAX38 without PASC as a cellulose matrix**. In these control reactions, related to Figure 3 in the main manuscript, WAX22 or WAX38 was incubated for 16 h at pH 6.5 and 40 °C with the GH62 enzyme only or together with the LPMO in the presence or absence of reductant. The control reactions without LPMO contained 0.5 μM CuSO_4_, to account for possible (copper-catalyzed) side reactions. Note that the WAX22 substrate contains low amounts of oligosaccharides, eluting in the 10 – 15 minutes region, which are negligible compared to the amounts of LPMO-generated WAX22-derived products seen in the reactions with both PASC and WAX22 that are depicted in Fig. 3 of the main manuscript.

**
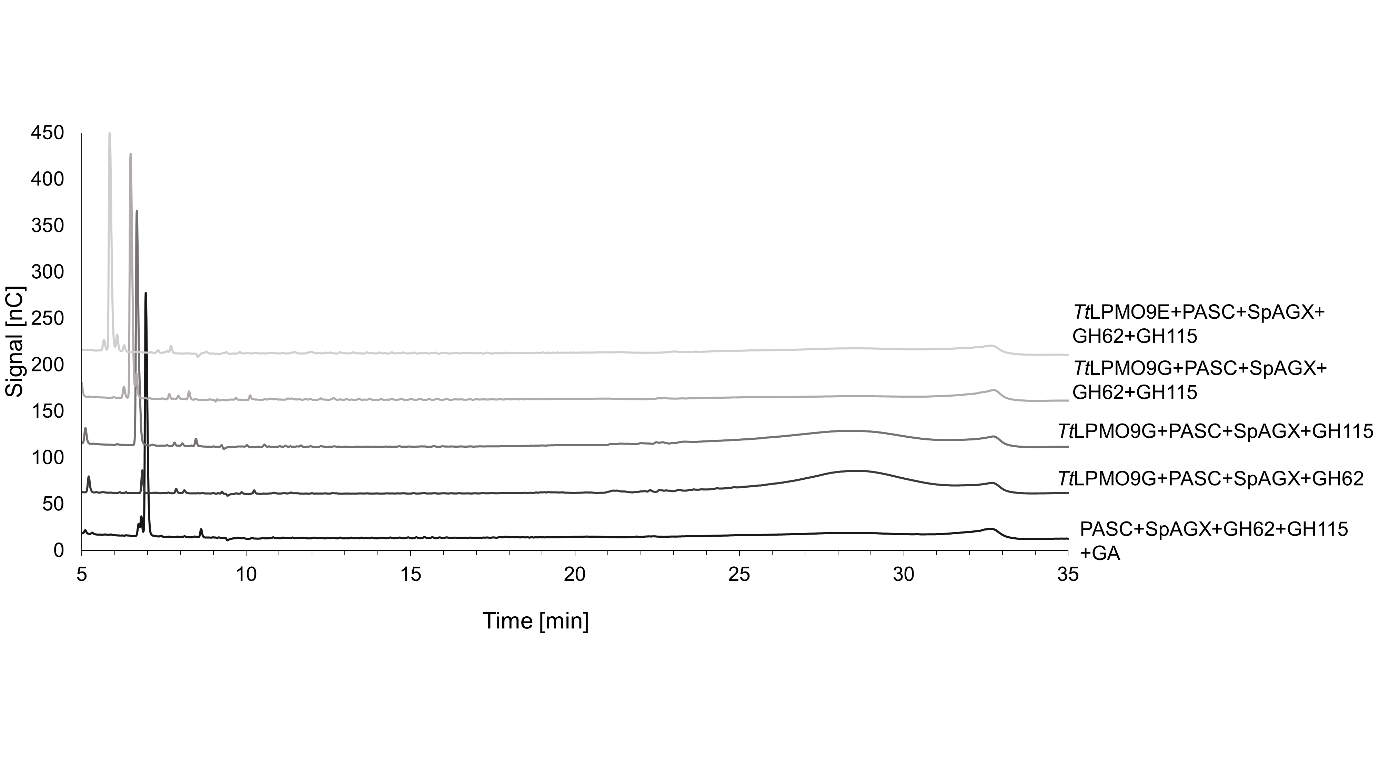
**

**Figure S4.** **HPAEC-PAD chromatograms for control reactions related to the assessment of LPMO9 activity on a mixture of PASC and SpAGX in the presence or absence of a *Um*GH62 and/or a *Bo*GH115A.** The control reactions are related to Figures 4a and 4b in the main manuscript. All reactions were set up in 50 mM BisTris-HCl buffer, pH 6.5, at 40°C for 16 h. In the reductant-free controls, GA was replaced with water and in the LPMO free reaction only 0.5 μM CuSO_4_ was added. The peak eluting at approximately 7 minutes likely represents methyl-glucuronic acid.


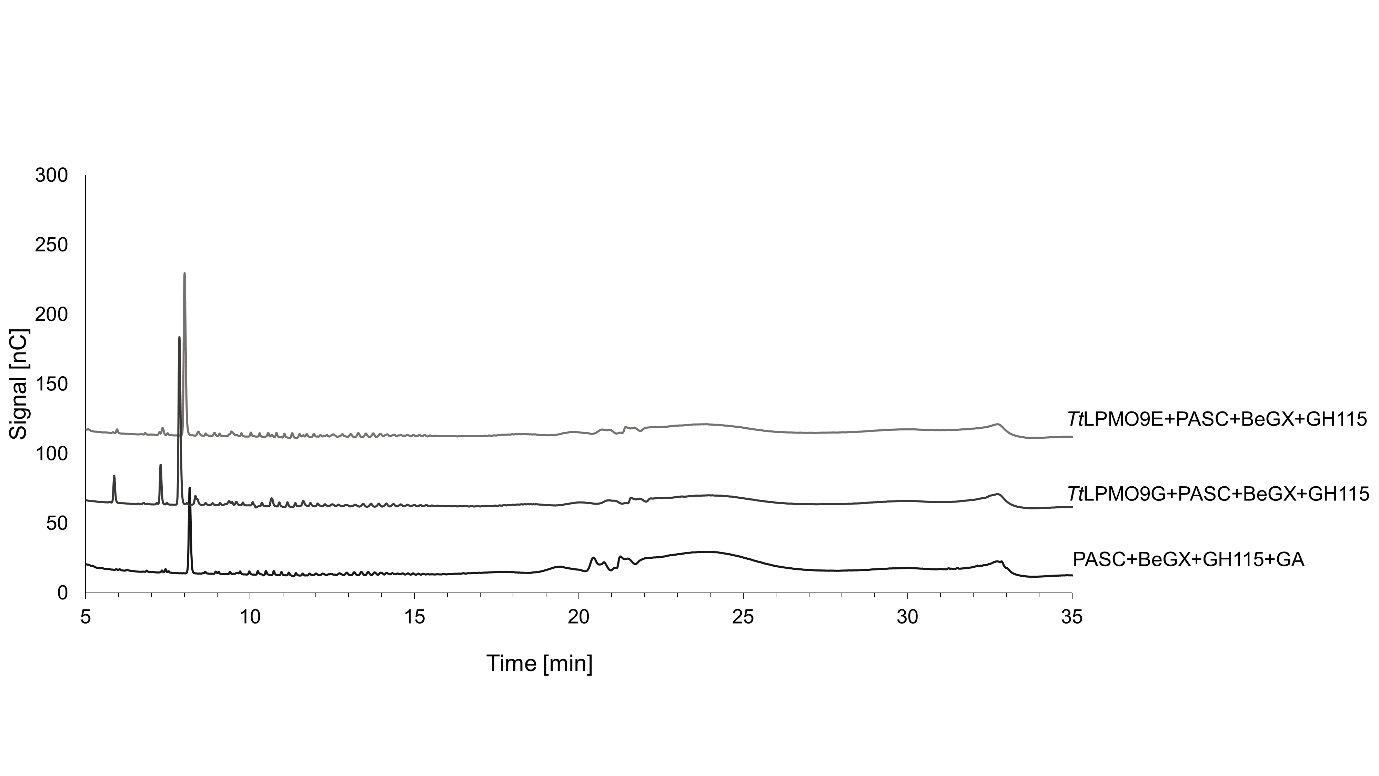


**Figure S5.** **HPAEC-PAD chromatograms for control reactions related to the assessment of LPMO9 activity on a mixture of PASC and BeGX in the presence or absence of *Bo*GH115A.** The control reactions are related to Figure 4c in the main manuscript. All reactions were set up in 50 mM BisTris-HCl buffer, pH 6.5, at 40°C, for 16 h. In the reductant-free controls, GA was replaced with water and in the LPMO free reaction only 0.5 μM CuSO_4_ was added. The peak eluting at approximately 7 minutes likely represents (methyl)-glucuronic acid.


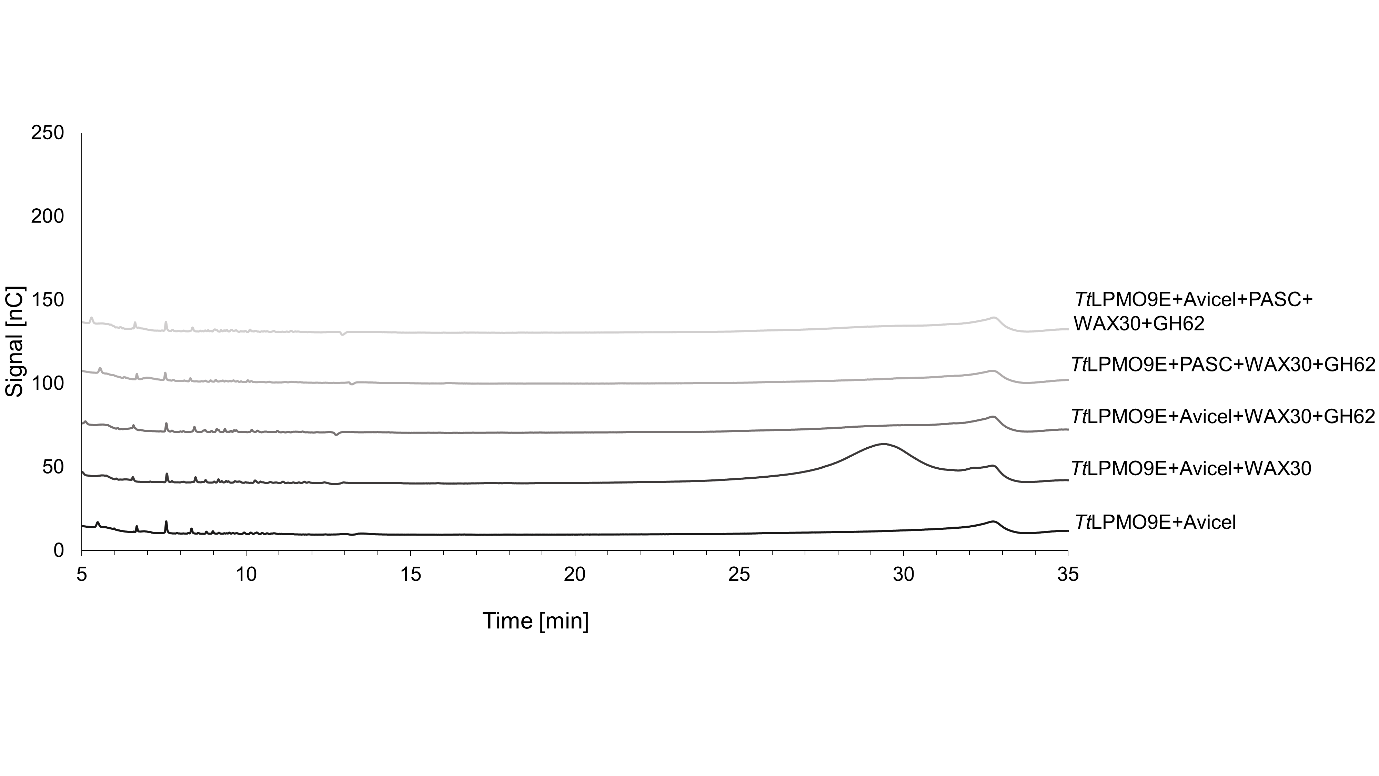


**Fig S6. HPAEC-PAD chromatograms for control reactions related to the assessment of *Tt*LPMO9E activity on WAX30 in the presence of PASC and/or Avicel and in the absence or presence of *Um*GH62.** The control reactions are related to Figure 6 in the main manuscript. In these controls, the reductant GA was replaced with water. The reactions were set up in 50 mM BisTris-HCl, pH 6.5, at 40°C, for 16 h.
